# Resting state EEG Abnormalities and their Relationship with TMS‐EEG induced Cortical Excitability in Alzheimer's Dementia

**DOI:** 10.1002/alz.088412

**Published:** 2025-01-09

**Authors:** Hiba Alhabbal, Gifty Asare, Renee P Lawson, Hamed Azami, Reza Zomorrodi, Robert Chen, Daniel M. Blumberger, Benoit H. Mulsant, Bruce G. Pollock, Tarek K. Rajji, Sanjeev Kumar

**Affiliations:** ^1^ Adult Neurodevelopment and Geriatric Psychiatry Division, CAMH, Toronto, ON Canada; ^2^ Institute of Medical Science, University of Toronto, Toronto, ON Canada; ^3^ IQVIA, Toronto, ON Canada; ^4^ Temerty Centre for Therapeutic Brain Intervention, CAMH, Toronto, ON Canada; ^5^ Krembil Research Institute, University of Toronto, Toronto, ON Canada; ^6^ Department of Psychiatry, Temerty Faculty of Medicine, University of Toronto, Toronto, ON Canada; ^7^ Temerty Centre for Therapeutic Brain Intervention CAMH, Toronto, ON Canada; ^8^ University of Toronto, Toronto, ON Canada; ^9^ Toronto Dementia Research Alliance, University of Toronto, Toronto, ON Canada; ^10^ Temerty Faculty of Medicine, Division of Psychiatry, University of Toronto, Toronto, ON Canada; ^11^ Temerty Faculty of Medicine, University of Toronto, Toronto, ON Canada

## Abstract

**Background:**

Previous literature has identified slowing of resting state electroencephalography (EEG) rhythm and abnormal cortical excitation in Alzheimer’s Dementia (AD). However, the relationship between these two divergent functional abnormalities and cognitive symptoms of AD are not well understood.

**Method:**

Resting state EEG signal was recorded in participants with AD and HCs for 5 minutes with eyes closed. Relative resting state EEG power was measured for the five frequency bands. Participants underwent a single pulse transcranial magnetic stimulation (TMS) combined with EEG. Cortical evoked activity (CEA) was assessed using TMS‐evoked potential (TEP) rectified area under the curve (AUC) from 25 to 80 ms post‐TMS stimulus, and the TEPs peak amplitudes were calculated by taking the maximal peak in the following time windows: 25 – 35 ms (P30), 40 – 50 ms (N45), and 55 – 65 ms (P60).

**Result:**

Compared to 32 HC (18 females; mean ± SD age: 69.3 ± 7.9 years), 52 participants with AD (32 females; 74.2 ± 8.5 years) had higher relative theta power than HCs (t(66.6) = 5.34, p<0.001). AD participants also had a significantly lower alpha power (t(76) = ‐3.03, p=0.003) and beta power (t(76) = ‐2.51, p=0.014) than HCs. Controlling for sex, age and years of education, AD participants showed a positive association between theta power and CEA (r_partial_=0.573, p=0.008); and an inverse association between alpha power and CEA (r_partial_=‐0.471, p=0.036). Theta power in AD participants showed a positive association with P60 (r_partial_=‐0.637, p=0.003) and an inverse association with N45 (r_partial_=‐0.512, p=0.025), while alpha power was inversely associated with P60 (r_partial_=‐0.531, p=0.016).

**Conclusion:**

This study showed a positive association between resting state EEG slowing and increased cortical excitability in AD, indicating a possible shared mechanistic pathway between these abnormalities.

